# An Exploratory Evaluation of GPT-5 in Periodontitis Staging and Grading Using Published Clinical Cases

**DOI:** 10.21203/rs.3.rs-8742868/v1

**Published:** 2026-02-03

**Authors:** Ihunna Amugo, Katie L. Frederickson, Harshana Rajakaruna, Hua Xie, Pandu Gangula, Anil Shanker, Qingguo Wang

**Affiliations:** Department of ODS & Research, School of Dentistry, Meharry Medical College, Nashville, TN 37208, USA; Department of Biochemistry, Cancer Biology, Neurosciences and Pharmacology, Meharry Medical College, Nashville, TN 37208, USA; The Office for Research and Innovation, Meharry Medical College, Nashville, TN 37208, USA; Department of ODS & Research, School of Dentistry, Meharry Medical College, Nashville, TN 37208, USA; Department of ODS & Research, School of Dentistry, Meharry Medical College, Nashville, TN 37208, USA; The Office for Research and Innovation, Meharry Medical College, Nashville, TN 37208, USA; Department of Biochemistry, Cancer Biology, Neurosciences and Pharmacology, Meharry Medical College, Nashville, TN 37208, USA

**Keywords:** large language model, ChatGPT, GPT-5, dental care, dental education, periodontitis, periodontitis staging and grading

## Abstract

**Background:**

Periodontitis is a chronic gum disease affecting approximately 42% of adults aged 30 and older in the United States. Training dental students to accurately diagnose and manage periodontitis is a critical component of dental education and clinical care. Recent advances in large language models (LLMs) offer new opportunities to support both domains, yet their performance in periodontal diagnosis remains largely unexplored—particularly for newer models such as Generative Pre-trained Transformer 5 (GPT-5).

**Objective:**

This study conducted an exploratory evaluation of GPT-5’s ability to stage and grade periodontitis.

**Methods:**

Twenty-five publicly available clinical cases were identified through Google and PubMed searches. Each case description was entered into GPT-5 using a zero-shot prompting approach, and the model’s predictions were compared with the published reference diagnoses. Performance was measured using accuracy and Cohen’s kappa.

**Results:**

Across these cases, GPT-5 showed marked class-dependent performance and a tendency to overestimate disease severity. Compared with prior models, it achieved comparable or improved performance, with accuracies of 68.0% for staging and 77.3% for grading and corresponding Cohen’s kappa values of 0.432 and 0.179, respectively. While staging performance showed fair agreement beyond chance, the low kappa for grading indicates poor agreement and limited reliability in distinguishing periodontal disease severity.

**Conclusions:**

These findings suggest that although GPT-5 shows improvement over previous models, its current diagnostic performance, particularly for periodontitis grading, limits its utility in clinical assessment and educational training. Meaningful application in periodontal diagnosis and training will require substantial improvements in reliability and rigorous validation. The limitations of the study and implications for future development are also discussed.

## Introduction

As the demand for accessible, accurate, and cost-effective resources to support dental care and education continues to grow, large language model (LLM)–based chatbots, such as Chat Generative Pre-trained Transformer (ChatGPT)[1], have emerged as promising tools. Although not originally developed for dental and healthcare applications, these systems can generate human-like responses with remarkable accuracy on many health-related topics [2–8], offering new opportunities for disease surveillance, biomedical research, and education.

Compared with traditional resources, LLMs offer distinct advantages for education and diagnostic support, including lower cost, continuous availability without the need for appointments, good accuracy for many diseases and conditions, customizable interactions, and user-friendly interfaces. As a result, people increasingly turn to them for medication information, self-diagnosis, and disease-prevention guidance[9–12]. Clinicians, dental and medical students also use them to acquire knowledge and support clinical decision-making[13, 14].

A growing body of research has examined LLMs’ utilities in dental care and education[5, 15–18]. Several benchmark studies have demonstrated LLMs’ competitive performance on the American Academy of Periodontology (AAP) in-service examination[6], the United States Medical Licensing Examination (USMLE)[2, 7], and other major assessments[1, 3, 8]. In an educational study involving 77 second-year dental students, those who used LLMs for learning assignments were found to perform better on knowledge examinations than peers relying on traditional methods[19]. Furthermore, Rahad *et al*. showed that ChatGPT excelled in recognizing and correcting specialized dental terminology and achieved 66.7% accuracy in extracting and synthesizing information from documents [5]. In the clinical context, Eroglu *et al*. evaluated ChatGPT-3.5 on 200 untreated periodontitis cases and reported moderate performance for staging and grading[4].

Despite these advances, critical gaps remain. Most prior studies used non-public datasets, limiting the reproducibility of their findings. Moreover, given the continual iteration and rapid improvement of LLMs, earlier assessments may not accurately reflect the capabilities of newer models such as Generative Pre-trained Transformer 5 (GPT-5), released in August 2025, whose performance in dentistry has not yet been systematically evaluated. Evaluation of LLMs in high-stakes dental clinical contexts is essential to establish quality-control mechanisms, mitigate risks of inaccurate or biased outputs, and guide their safe adoption into dental education and care.

An important component of dental education and care is training students to diagnose and manage periodontitis, a chronic gum disease affecting approximately 42% of adults aged 30 and older in the US[20]. Periodontitis staging (I–IV) reflects disease severity and extent based on levels of destroyed tissues, including gingival attachment and alveolar bone, while grading (A–C) estimates the rate of progression and future risk[21–23]. Even with explicit and standardized criteria for staging and grading[21–23], clinical diagnosis of periodontitis remains challenging and context-dependent, requiring careful integration of radiographic evidence, periodontal charting, and patient-specific risk factors. An LLM capable of accurately analyzing, staging, and grading clinical periodontitis cases could serve as both a valuable diagnostic aid for clinicians and a useful educational resource for students. To address this need, we conducted an exploratory evaluation of the performance of the newly released GPT-5 in staging and grading periodontitis cases.

## Methods

### Case Identification and Data Collection

We identified dental clinical cases by searching Google and PubMed in August and September 2025 using the keywords ‘periodontitis’, ‘staging’, and ‘grading.’ These searches targeted peer-reviewed articles, case reports, and publicly available teaching materials that explicitly described periodontitis staging and grading according to established clinical criteria.

**Figure 1:**
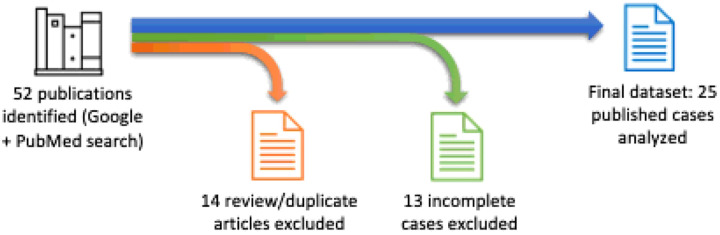
Data collection and case selection workflow.

All records retrieved from the searches were screened manually. Review articles, duplicate records, and reports lacking complete staging or grading diagnoses, sufficient periodontal clinical descriptions, or adequate medical and dental histories were excluded. After screening, 25 cases were retained from a total of 52 identified records. The workflow for data collection and case selection is illustrated in Fig. 1.

Most of the public cases collected included panoramic and periapical radiographs, which, together with patients’ dental histories and periodontal charting, play a central role in diagnosis. In these cases, the radiographs had already been systematically evaluated by the original authors, and numerical measures of the bone loss were extracted and reported in their publications. We manually extracted these clinically meaningful data from the publications and used them directly in our assessments. Radiographic images themselves were not used in our analyses.

It is important to note that publicly available and teaching-oriented cases, which are often unusually well documented and may not fully reflect the spectrum of routine clinical presentations, may introduce selection bias and limit generalizability. As a result, the GPT-5 performance observed in this exploratory study should be interpreted cautiously and may not reflect real-world diagnostic performance. These limitations are discussed in greater detail in the Discussion section.

### GPT-5 Prompting and Evaluation

While GPT-5 can process multimodal data, we used only its interactive textual interface. All analyses were conducted between September 1st and 12th, 2025, using the free GPT-5 version, without any paid enhancements. GPT-5 was accessed through its publicly available interactive interface, which does not permit user control over system-level parameters such as temperature and system prompts. Therefore, all interactions were conducted using the default system configuration (temperature = 1.0).

Given GPT-5’s demonstrated accuracy and reasoning improvements over earlier models [24], and because the criteria for periodontitis staging and grading outlined in the 2017 World Workshop are strictly guideline-based [21–23], we adopted a zero-shot prompting strategy. This approach evaluates GPT-5’s ability to apply explicit clinical thresholds and decision logic without influence from exemplar conditioning. In contrast to few-shot prompting or fine-tuning [25–27], which may introduce anchoring effects or label leakage and thereby inflate performance, zero-shot prompting provides a more conservative and transparent assessment of model reasoning.

Using zero-shot prompting, case descriptions were submitted directly to the model without examples or prior instructions, and GPT-5 was asked to return predictions of periodontitis stage and grade. Before submission, case descriptions were lightly reformatted to correct line breaks, spacing, and formatting artifacts introduced during PDF extraction to improve readability for the model. No clinical content was added, removed, reworded, or reorganized, and the original meaning and structure of the source material were preserved. An example prompt for a clinical case and a detailed ChatGPT response are provided in the appendix.

To assess response stability, each case was tested in multiple sessions using slight variations in prompt phrasing (e.g., “Can you help determine the periodontitis stage and grade of this patient?” or “Please stage and grade the periodontitis of this patient”). The model consistently generated identical predictions, regardless of prompt phrasing or session timing, indicating stable behavior at the decision level under these conditions. For the final analyses reported in this study, we used a standardized prompt, “Please stage and grade the periodontitis of this patient”, followed by the corresponding clinical case description.

Data collection and analysis were conducted by four domain experts, all of whom are co-authors of this study. The team included a dental student, two professors from the School of Dentistry, and a professor of Data Science from the School of Medicine at Meharry Medical College. Their responsibilities included extracting clinical cases from publications, developing prompts for GPT-5, and verifying the model’s responses.

### Performance Metrics

GPT-5 predictions were compared against published ground-truth diagnoses to calculate model accuracy and Cohen’s kappa. To characterize error patterns, confusion matrices were generated for both staging and grading to allow assessment of tendencies toward overestimation or underestimation. Given the limited dataset size (n = 25) and the exploratory nature of this study, results are presented as descriptive performance metrics, without formal hypothesis testing. All data analyses were conducted in RStudio (v2025.09.2), and visualizations were generated using the R package ggplot2 (v4.3.3).

## Results

### Description of Periodontitis Cases

Of the 25 periodontitis cases collected for evaluating GPT-5, two were borderline cases with staging ambiguities between Stage III and Stage IV, and three provided only staging information in the original publications. Full case descriptions and corresponding sources are provided in the Appendix.

**Figure 2:**
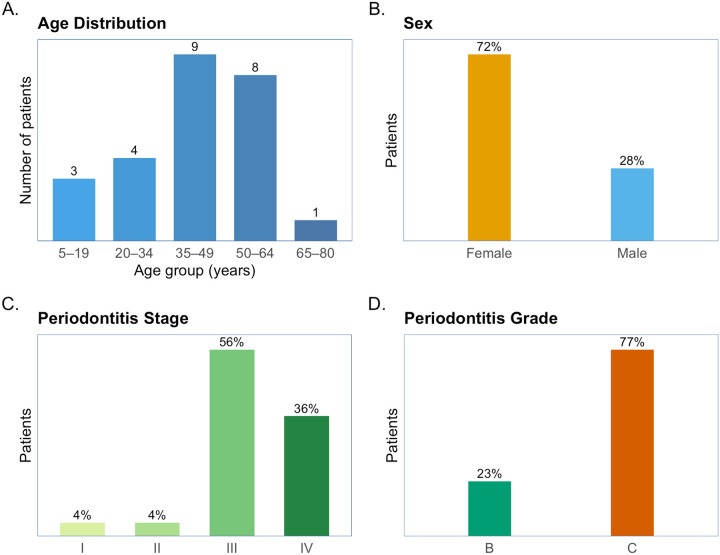
Demographic and clinical characteristics of 25 patients with periodontitis.

Figure 2 summarizes the cohort. The median age of these patients is 45 years, with most cases (68%) occurring between 35 and 64 years (Fig. 2A). Females comprised the majority (72%), while males accounted for 28% (Fig. 2B). In terms of disease severity, Stage III periodontitis was most common (56%), followed by Stage IV (36%), Stage II and I (4%) (Fig. 2C). For grading, most cases were classified as Grade C (77%), with smaller proportions in Grade B (23%) (Fig. 2D).

### Workflow for evaluating GPT-5

**Figure 3:**
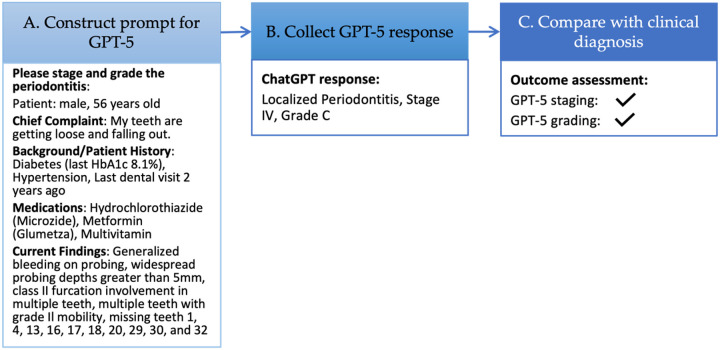
Framework for evaluating GPT-5 performance in periodontitis staging and grading. (**A**) A prompt was constructed from a publicly available case[28], with minor formatting adjustments (e.g., line breaks and spacing) to improve readability for the model while preserving the original content. (**B**) GPT-5 generated categorical outputs for periodontitis stage (I–IV) and grade (A–C). (**C**) Model predictions were compared with the clinical reference diagnoses reported in the original publication to assess accuracy.

Figure 3. Framework for evaluating GPT-5 performance in periodontitis staging and grading. (**A**) A prompt was constructed from a publicly available case[28], with minor formatting adjustments (e.g., line breaks and spacing) to improve readability for the model while preserving the original content. (**B**) GPT-5 generated categorical outputs for periodontitis stage (I–IV) and grade (A–C). (**C**) Model predictions were compared with the clinical reference diagnoses reported in the original publication to assess accuracy.

The prompt was then submitted to GPT-5, which was asked to determine the stage and grade of periodontitis. The model’s response was collected (Fig. 3B), recording only the predicted stage and grade while disregarding the diagnostic reasoning. The output was compared against the clinical diagnosis to evaluate accuracy (Fig. 3C).

After all cases were processed, GPT-5’s predictions were aggregated, and performance metrics were calculated to summarize its diagnostic accuracy and Cohen’s kappa and assess its potential utility in real-world clinical settings.

### GPT-5 performance in periodontitis staging and grading

Across the 25 periodontitis cases (including the two borderline cases), GPT-5 achieved 68.0% accuracy for staging (17/25 correct) and 77.3% accuracy for grading (17/22 correct, excluding three cases without specified grades). The corresponding Cohen’s kappa values were 0.432 for staging and 0.179 for grading, indicating fair agreement and poor agreement beyond chance, respectively.

**Figure 4:**
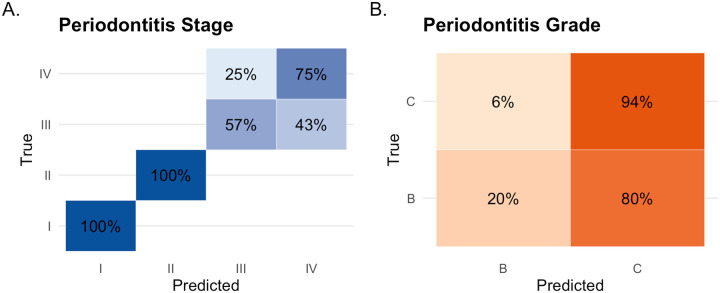
Confusion matrices of GPT-5 predictions for periodontitis staging and grading.

The confusion matrix in Fig. 4A shows that all Stage I and II cases were classified correctly (recall = 100%), whereas recall was substantially lower for Stage III (57%) and Stage IV (75%). For grading, performance was markedly imbalanced, with high recall for Grade C (94%) but very low recall for Grade B (20%) (Fig. 4B). This class-dependent performance indicates that GPT-5 performs well for early-stage periodontitis and advanced disease detection but struggles to reliably distinguish intermediate disease categories.

Importantly, misclassifications exhibited nonrandom patterns: errors were confined to adjacent categories, with no Stage I or II cases misclassified as Stage III or IV, no Stage IV cases misclassified as Stage I or II (Fig. 4A), and no “skipping” across nonadjacent categories for grading (Fig. 4B). Because all errors were limited to adjacent stages and grades and no catastrophic misclassifications were observed, these patterns are clinically and educationally relevant.

Among the misclassified cases, 43% of Stage III cases were predicted as Stage IV, while 25% of Stage IV cases were predicted as Stage III (Fig. 4A). For grading (Fig. 4B), GPT-5 correctly classified 94% of Grade C cases, with most Grade B cases misclassified as Grade C. These results suggest that GPT-5 tends to assign higher severity for both periodontitis stage and grade. GPT-5’s predictions for the 25 cases are provided in the Appendix.

**Figure 5:**
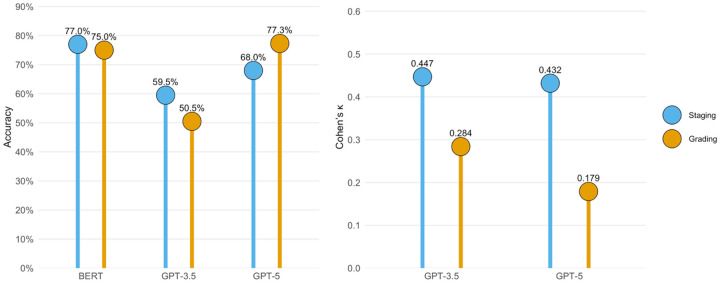
Comparison of BERT, GPT-3.5, and GPT-5 performance in periodontitis staging and grading. Data are derived from Table 1.

Table 1 and Fig. 5 compare our findings with two prior assessments. Eroglu *et al*. evaluated GPT-3.5, an earlier ChatGPT version, on 200 untreated patients and reported 59.5% accuracy in staging and 50.5% accuracy in grading[4]—both notably lower than the performance achieved by GPT-5 on our dataset. The low kappa values observed for both models (0.284 for GPT-3.5 and 0.179 for GPT-5) in Table 1 underscore ChatGPT’s limited discriminatory ability in grading periodontitis beyond chance agreement.

Another study summarized in Table 1 and Fig. 5, conducted by Ameli *et al*. [29], fine-tuned a Bidirectional Encoder Representations from Transformers (BERT) model using 309 anonymized periodontal charts and corresponding clinician notes. The model was trained on 70% of the data and tested on 32 holdout cases. The fine-tuned BERT model achieved 77.0% accuracy in staging and 75.0% in grading[29]. Although it outperformed GPT-5 in staging, it was slightly inferior in grading despite being specifically optimized for periodontitis. This comparison should be interpreted with caution, however, as BERT was evaluated on periodontal charts and clinician notes, whereas GPT-5 and GPT-3.5 were assessed using standardized textual case descriptions that included patient age, sex, and numerical measures of periodontitis-related parameters.

## Discussion

### Limitations

Our exploratory evaluation of GPT-5 relied solely on published cases, which represent a relatively limited sample. Moreover, both the small sample size and the gender imbalance within the data may disproportionately reflect more severe or well-documented presentations, potentially inflating GPT-5’s performance. In addition, publicly available case reports are often curated to highlight clear diagnostic features and may not accurately reflect the full clinical heterogeneity or noise encountered in real-world practice. As a result, such cases may underrepresent diverse patient populations and disease presentations commonly seen in routine clinical settings. Expanding the dataset in future studies to include larger, more diverse, and non-published clinical data will therefore be essential to broaden the scope of evaluation, strengthen generalizability, and support meaningful clinical translation.

Additional methodological constraints include our use of GPT-5’s interactive interface, which does not allow modification of underlying system parameters such as temperature, and hence may restrict reproducibility at the system level. Moreover, this study did not evaluate model stability across alternative prompting strategies, which are known to influence LLM behavior. Furthermore, only two borderline or equivocal cases were included, which are insufficient to assess GPT-5’s performance in diagnostically challenging scenarios where clinician disagreement is common and clinical judgment plays a critical role. Future studies should therefore incorporate more diagnostically ambiguous cases and systematically compare prompting strategies to better evaluate model reliability and clinical relevance.

Although this study focused narrowly on GPT-5’s performance in periodontitis staging and grading, the potential applications of LLMs extend more broadly to both clinical diagnostics and dental education. In clinical practice, LLMs could assist practitioners by consistently applying standardized staging and grading criteria, integrating charting and radiographic data, and generating preliminary assessments. In education, LLMs can function as personalized learning assistants, offering structured feedback on case analyses and helping students navigate diagnostic complexity. Beyond these applications, LLM-based chatbots hold promise for reducing gaps in dental educational resources, particularly in under-resourced institutions, thereby strengthening their capacity to deliver high-quality dental education and care.

However, the observed diagnostic agreement, particularly for periodontitis grading, highlights an important limitation. The low Cohen’s kappa (κ = 0.179) for grading indicates poor agreement beyond chance, suggesting that GPT-5 currently lacks sufficient reliability to accurately distinguish between periodontitis grades. Consequently, GPT-5 is not yet suitable for independent clinical grading, and its outputs should be interpreted with caution. In addition, in the absence of established minimum clinically important difference (MCID) thresholds for AI-assisted periodontitis staging/grading, κ = 0.432 for staging should be interpreted cautiously and not as evidence of clinical readiness.

Looking forward, GPT-5 and other LLMs are likely to continue to improve diagnostic performance. Nevertheless, its meaningful clinical translation will hinge on overcoming current deficiencies in reliability and consistency. Beyond advancements in model development, a potential pathway toward high-stakes clinical and educational applications likely lies in the integration of LLMs with validated AI tools optimized specifically for clinical use. Such hybrid systems, which combine the precision of specialized diagnostic models with the reasoning, interpretability, and interactivity of LLMs, may provide more robust support for complex, multimodal clinical decision-making in dental care and education.

Finally, this exploratory study was conducted with consideration of established AI reporting guidelines, including DECIDE-AI and STROBE-AI [30–32]. While the exploratory design and reliance on publicly available clinical cases, rather than real-time clinical data, precluded full adherence to all framework components, key principles such as transparency in data sources, model usage, limitations, and reproducibility were followed. Future prospective studies using large-scale, real-world clinical data will be better positioned to fully implement these reporting standards.

### Conclusions

With the growing use of LLMs by dental and medical students, clinicians, and the general public, it is important to evaluate their performance in high-stakes diagnostic and educational settings to inform safety protocols and guide responsible applications. In this study, we assessed GPT-5’s ability to stage and grade periodontitis—tasks central to periodontal diagnosis and student training. Compared with prior models, GPT-5 demonstrated comparable or improved performance, achieving 68.0% staging accuracy and 77.3% grading accuracy, with a staging kappa of 0.432, indicating fair agreement beyond chance.

However, despite these performance gains, the low kappa for grading (0.179) underscores the very limited discriminatory capacity in distinguishing periodontitis grades, indicating that GPT-5 is not yet suitable for independent clinical applications. Additionally, our results revealed a consistent tendency for GPT-5 to overestimate disease severity. Therefore, inappropriate reliance on model outputs could increase the risk of overtreatment or unnecessary escalation of care. This underscores the importance of human oversight and the need for future evaluations of uncertainty reporting, refusal behavior, and decision-level safeguards before any clinical integration is considered.

In conclusion, while GPT-5 demonstrated potential as a supportive tool for education and clinical exploration, it is not yet ready for autonomous use. Meaningful application in periodontal diagnosis and training will depend on substantial improvements in reliability and rigorous validation.

## Figures and Tables

**Table 1:** Recent studies of periodontitis staging and grading using textual input.

Dataset^+^	Model	Accuracy		Cohen’s kappa		Study ID
		Stage	Grade	Stage	Grade	
309 periodontal charts and clinical notes	BERT	77.0%	75.0%	NA	NA	Ameli *et al*.
200 untreated periodontitis patients	GPT-3.5	59.5%	50.5%	0.447	0.284	Eroglu *et al*.
25 dental clinical cases from public sources	GPT-5	68.0%	77.3%	0.432	0.179	Our result

+ The findings from the first two studies were extracted from published papers, while the details of our own assessment results are provided in the Appendix.

## Data Availability

The sources of our dental clinical cases are provided in the Appendix.

## Multimedia Appendix 1

GPT-5’s performance in periodontitis staging and grading on the textual input of 25 clinical cases.

## Abbreviations

BERT: Bidirectional Encoder Representations from Transformers
GPT: Generative Pre-trained Transformer
LLM: Large language model
